# Evaluation of plasma ACTH in the metyrapone test is insufficient for the diagnosis of secondary adrenal insufficiency

**DOI:** 10.3389/fendo.2022.1004129

**Published:** 2022-11-10

**Authors:** Lucyna Papierska, Michał Rabijewski, Bartosz Migda, Dorota Leszczyńska, Karolina Nowak, Agnieszka Łebek-Szatańska, Piotr Glinicki, Wojciech Zgliczyński

**Affiliations:** ^1^ Department of Endocrinology, Medical Centre for Postgraduate Education, Warsaw, Poland; ^2^ Department of Reproductive Health, Medical Centre for Postgraduate Education, Warsaw, Poland; ^3^ Diagnostic Ultrasound Lab, Department of Pediatric Radiology, Medical Faculty, Medical University of Warsaw, Warsaw, Poland; ^4^ Department of Endocrinology, Bielański Hospital, Warsaw, Poland

**Keywords:** secondary adrenal insufficiency, metyrapone test, ACTH, short synacthen test, adrenal insufficiency

## Abstract

**Objective:**

To determine whether a single measurement of ACTH instead of less available in daily practice 11-deoxycortisol assay is sufficient to rule out or confirm secondary adrenal insufficiency (SAI) in the short Metyrapone test.

**Design:**

A retrospective analysis of diagnostic tests (Metyrapone and Synacthen tests) performed at our Center between 2016 and 2018 in patients with suspicion of secondary adrenal insufficiency.

**Material and methods:**

In 103 patients short metyrapone test was performed with assessment of 11-deoxycortisol and ACTH concentration after Metyrapone administered at midnight. In 89 of them short Synacthen (SST) test was also done (1 or/and 250 mcg 1-24ACTH). ROC curves have been performed to evaluate the diagnostic performance of ACTH level in metyrapone test as the predictor of secondary adrenal insufficiency (SAI) analysing sensitivity and specificity for various possible thresholds proposed in literature.

**Results:**

40 (39%) of examined subjects were diagnosed as SAI, basing on post-Metyrapone 11-deoxycortisol concentration below 70 μg/l. In this group ACTH concentration was 128.1 ng/l (95% CI 96.8-159.4) versus 289.9 ng/l (95% CI 249.1-330.9) in patients with proper adrenal response. There was only a moderate positive correlation between ACTH and 11-deoxycortisol concentrations (r=0.5; p<0.05). The best cut off value of ACTH in relation to 11-deoxycortisol serum concentrations was 147 ng/l - with sensitivity of 73.2% and specificity 83.9%. However, plasma ACTH was>200ng/ml (the highest threshold proposed in literature) in 8 cases (20%) with positive diagnosis of SAI made on the basis of low 11-deoxycortisole and confirmed in short Synacthen test.

**Conclusion:**

Our results indicate that for a valuable evaluation of the results of the metyrapone test, the more readily available plasma ACTH assay cannot replace the measurement of 11-deoxycortisol concentrations.

## Introduction

Diagnosis of adrenal insufficiency (AI) seems to be easy, however, in many cases it is stated only after adrenal crisis, despite obvious symptoms occurring for many months (1–4). Especially secondary forms of disease could be difficult to recognize, as signs and symptoms are non-specific and less pronounced in comparison to primary AI (5–7). On the other hand, misdiagnosed patients with relatively low basal cortisol levels but proper hypophyseal reserve of ACTH are exposed to unnecessary medication with hydrocortisone, which, even in substitutive doses, can increase cardiovascular risk (8, 9).

The most frequently used test in the diagnosis of adrenal insufficiency ACTH stimulation test is a valuable diagnostic tool for primary AI. In this procedure, called the short Synacthen test (SST), serum cortisol concentration is assessed before, 30 minutes, and 60 minutes after intravenous administration of 250 μg synthetic ACTH (1–24 corticotropin, Tetracosactide, Synacthen). Any value ≥ 18 μg/dl defines a proper response of healthy adrenal glands (10). Since after the injection of 250 μg 1-24 ACTH supra-physiological concentration of corticotrophin is achieved, a stimulation with 1 μg dose was proposed. Despite numerous studies, there is still no clear evidence that this variation of the test has an advantage over the “classic” one (11). However, in secondary adrenal insufficiency (SAI) before adrenal atrophy has occurred, the sensitivity of both tests may be too low to recognize the disease (6, 12). The insulin tolerance test is based on the fact that hypoglycemia is one of the most serious physiological stresses which therefore stimulates hypophysis to release a great amount of ACTH and consequently causes an increment in serum cortisol level. This procedure, causing hypoglycemia, is neither pleasant nor safe for the patient, requires physician supervision, moreover is contraindicated in many medical conditions (e.g. in patients with ischemic heart disease, cerebrovascular disease, seizures) (13, 14).

CRH stimulation tests also give false negative results (FN), so can’t be recommended as a first-line diagnostic tool (15, 16).

Some clinical centers, including ours, prefer metyrapone tests for the diagnosis of secondary adrenal insufficiency (15–19). The essence of this test is an assessment of the hypophyseal (and consequently adrenal) response to drug-blocking 11 beta-hydroxylase, an enzyme required for the transition from 11-deoxycortisol to cortisol. The administration of metyrapone (in single or repeated doses) causes a decrease in blood cortisol concentration, further incrementing ACTH, and finally the arousal of adrenal steroids synthesis, ending with 11-deoxycortisol, whose level is measured during the test (20). However, the measurement of 11-deoxycortisol concentration is not a standard procedure in most laboratories. Since the essence of the Metyrapone test is to evaluate the corticotropin reserve of hypophysis, the assessment of the ACTH increment would be the ideal single measurement to state the diagnosis. However, recommendations in different guidelines regarding the interpretation of ACTH results in this test are inconsistent. Proposed cut-off values for post-metyrapone ACTH response vary from >75 ng/l (21) by 150 ng/l (22) to >200 ng/l (23).

The aim of the study was the evaluation of ACTH response to single-dose Metyrapone administered at midnight and a comparison of ACTH and 11-deoxycortisol concentrations achieved during the test.

## Material and methods

### Patients

Data of all patients who underwent a metyrapone test in our tertiary endocrinology center (Department of Endocrinology, Centre of Postgraduate Medical Education, Warsaw, Poland) from January 2016 to December 2018 were collected retrospectively. The reason for the test was the suspicion of secondary adrenal insufficiency stated by general practitioners or endocrinologists in the primary center. Patients were tested by us if previous baseline test results were inconclusive: cortisol levels between 5 and 10 µg/dl in at least two determinations. Since many centers in Poland have problems with ACTH measurement (inadequate sample processing), we did not include results of ACTH concentrations obtained outside our center in the qualification for further studies. If the patient was currently treated with hydrocortisone, the medication was stopped 2 days before the hospitalization.

### Metyrapone test

Metyrapone (Metopirone, HRA Pharma Rare Diseases) was administered at midnight in a dose of 30 mg/kg b.w., swallowing with one glass of water; a little snack was also allowed if necessary. Levels of plasma ACTH, serum cortisol, and serum 11- deoxycortisol were measured the next morning, at 8:00 am. The blood samples were collected in a prone position, and 20 mg of Hydrocortisone was taken by the patient immediately after blood donation. Serum cortisol below 5 μg/dl was considered an adequate inhibition of 11 β-hydroxylase.

Additionally, in 89 cases the short Synacthen test (SST) test was performed with 1μg (low dose – LD-SST) or 250μg (high dose – HD-SST) intravenous Synacthen injection. The washout period between Metyrapone and Synacthen tests was 3-14 days. Both LD- and HD-Synacthen tests were carried out between 9:00 am and 10:00 pm. All tests were performed by the same group of well-trained medical personnel.

### Laboratory analysis

Serum cortisol concentration was determined using the chemiluminescent immunoassay (CLIA) using the UniCel DxI 600 analyzer (Beckman Coulter, UK). The analytical sensitivity was 0.4 µg/dl (11 nmol/l). The reference range for cortisol determined in the morning (7.00-9.00) is 6.7-22.6 µg/dl (185-624 nmol/l). Conversion factor: µg/dl x 27,8 = nmol/l.

Plasma ACTH concentration was determined, immediately after the blood donation, using the chemiluminescent immunoassay (CLIA) with the LIAISON XL analyzer (DiaSorin, Italy). The analytical sensitivity was 1.6 ng/l. The reference range for ACTH measured in the morning (7.00-9.00) is 4.7 - 48.8 ng/l. Conversion factor: ng/l x 0.2202 = pmol/l.

Sera for assessment of 11-deoxycortisol levels were frozen at -86°C (as we don’t have the ability to perform this analysis day-to-day). The determination of the serum 11-deoxycortisol was performed using the radioimmunoassay (RIA) method with kits from Diasource ImmunoAssays (Belgium). The analytical sensitivity was 0.11 ng/ml. Expected values (reference ranges): determination of 11-DOC concentration under baseline conditions (without stimulation) is <0.255 μg/dl (<7.2 nmol/l), while in the level after stimulation with Metyrapon is 7.2 – 22.5 μg/dl (208-649 nmol/l). Conversion factor: 1 μg/dl = 0.2886 nmol/l.

### Statistical analysis

Statistical analyses were performed using Statistica software (version 13.1 Dell. Inc. Statsoft). Presented data were expressed as means with a 95% confidence interval. Data distributions were assessed by the W Shapiro-Wilk test. For the comparison of nonparametric single variables, the Mann-Whitney test was used. For nominal variables frequencies were calculated with sensitivity, specificity, positive and negative predictive values, accuracy, and risk ratio with a 95% confidence interval. Frequencies were compared using the Fisher exact test. Correlation between parametric and nonparametric data was assessed by Pearson or Spearman coefficient, respectively.

We performed receiver-operating characteristic (ROC) curves to evaluate the diagnostic performance of ACTH level in the metyrapone test as a predictor of secondary adrenal insufficiency (SAI) analyzing sensitivity and specificity for each possible threshold/cut-off, and we used the area under the ROC curve to express the overall diagnostic accuracy of the index criterion. We have reported also a 95% confidence interval for AUC and p-value. P < 0.05 was considered indicative of a statistically significant difference.

## Results

Out of the 103 study participants, 11 (10.6%) were males and 92 (89.4%) were females. The mean age was 48.5 years (range 25-88 years), women 48.4 years (range 25-83 years), men 49.4 years (range 29-88 years).

There were 14 patients who were more than 2 months after cessation of prolonged corticotherapy (treated for 6-36 months with 5-7.5 mg of Prednisone daily for rheumatoid arthritis or asthma), 8 were 6-24 months after pituitary neurosurgery, 7 had “empty sella” on MRI and 3 were 6,12 and 24 months after unilateral adrenalectomy due to hypercortisolaemia. The other causes of investigation for adrenal reserve were: hyponatraemia, hypoglycaemia, and hypotonia, muscle pains, lack of appetite, and loss of body weight. Average morning serum cortisol concentration before tests was 8,05 ±0,64 µg/dl and plasma ACTH level was 14,3 ±12,7 ng/l Basal serum cortisol levels were below 10 μg/dl in 75 subjects and in 45 basal plasma ACTH was below 10 ng/l.

### Metyrapone test

There were 40 subjects with post-Metyrapone 11-deoxycortisol concentration below 7 μg/dl, so they were classified as patients with confirmed adrenal insufficiency - AI(+). In 22 subjects of this group diagnosis of adrenal insufficiency was additionally confirmed by insufficient cortisol increment in the 30^th^ and 60^th^ minute of the Synacthen test (when we took into account only cortisol in the 30^th^ minute this number increased to 31). In group AI+ concentration of 11-deoxycortisol was 4.74 μg/dl. (95% CI 4.19-5.29) while in “healthy” group, i.e. with 11-deoxycortisol>7 μg/dl – AI(-) was 10.54 μg/dl (95% CI 9.91-11.19)

A decrease of serum cortisol concentration under 5 μg/dl is considered a positive reaction to the test with metyrapone. Mean value of cortisol was 3.2 μg/dl (95% CI 2.9-3.5), whereas in subgroup SAI(+) and SAI(-) the values significantly differ 2.4 μg/dl (95% CI 2.0-2.9) vs 3.7 μg/dl (95% CI 3.4-3.9).

The concentration of post-Metyrapone plasma ACTH for the whole group was 225.6 ng/l (95% CI 194.3-256.9), whereas in group A it was lower: 128.1 ng/l (95% CI 96.8-159.4) vs 289.9 ng/l (95% CI 249.1-330.9) (**Table 1**).

**Table 1:** Metyrapone test.

Analyzed group	Mean (95% Cl)	Min	Max	Negative	Positive	p-value
N =103				Mean (95% Cl)	Mean (95% Cl)	
11-Deoxycortisol, µg/dl	8.24 (7.53-8.94)	0. 075	17.6	4.74 (4.19-5.29)	10.54 (9.91-11.19)	<0. 0001
ACTH, ng/l	225.6 (194.3-256.9)	2.0	804	128.1 (96.8-159.4)	289.9 (249. 1-330. 9)	<0. 0001
Cortisol, µg/dl	3.2 (2.9-3.5)	0.1	5	2.4 (2.0-2.9)	3.7 (3.4-3.9)	<0. 0001

95% CI, 95% confidence interval; p-value for U Mann-Whitney-test.

There was only a moderate positive correlation between ACTH and 11-deoxycortisol concentrations (Pearson r=0.5; p<0.05) and a moderate negative correlation between ACTH and cortisol level in the metyrapone test (Pearson r= - 0.3; p<0.05). There were no significant differences for this calculation in the analysed subgroups.

Analysis using ROC curves revealed that the best cut-off value of post-Metyrapone ACTH in relation to 11-deoxycortisol concentrations was < 147 ng/l - with a sensitivity of 73.2% (predicting SAI) and specificity 83.9% (positive SAI in case of 11-deoxycortisol level under 7.0 µg/dl, negative SAI when 11-deoxycortisol concentrations were at least 7.0 µg/dl). (AUC 0.833; 95% CI 0.749 – 0.917; p=0.00001). The computed risk ratio (RR) was 4.3 (95% CI 1.9 – 9.5), which means that patients with post-Metyrapone ACTH concentration lower than 147 ng/l had 4.3 times the risk of SAI compared to the group of patients who had ACTH level at least 147 ng/l.

### Synacthen test

In the beginning of this analysis we have compared cortisol concentrations levels 30 and 60 minutes after Synacthen administration comparing mean values between LD-SST and HD-SST. There was no statistically significant difference (p>0.05) and in further analyses, we treated both types of the test as equivalent (**Table 2**).

**Table 2:** Difference of cortisol level between LDSST and HDSST after 30’ and 60’.

		N	Mean (95% Cl)	Max	p-value
30 minutes			18.4 (17.2-19.7)	34.1	0.1823
	LDSST	30	20.0 (18.2-21.8)	34.1	
	HDSST	59	17.6 (16.0-19.2)	32.6	
60 minutes			21.2 (19.6-22.8)	40.7	0.1151
	LDSST	30	18.9 (17.9-22.4)	40.5	
	HDSST	59	21.8 (19.6-23.9)	40.7	

95% CI, 95% confidence interval; p-value for U Mann-Whitney test; cortisol valuesgiven in µg/dl.

**Figure 1:**
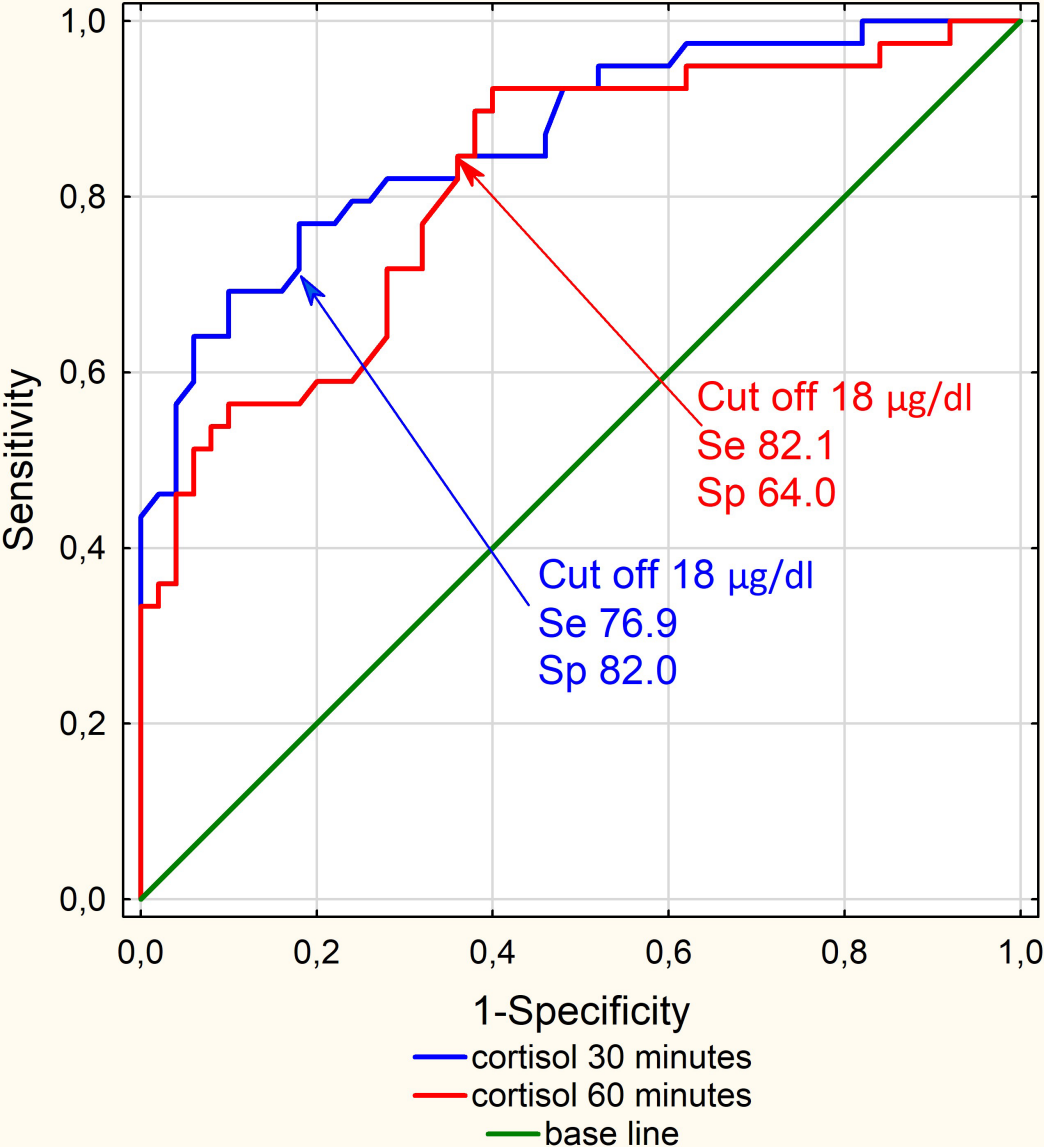
ROC curves for Cortisol after Synacthen administration - 30 minutes (AUC 30’ 0.865; 95% CI 0.788 – 0.941) in relation to 60 minutes (AUC 60’ 0.814; 95% CI 0.725 – 0.904) N=89.

The cortisol concentration increased from baseline to 30 and 60 minutes after injection of Synacthen (p=0.0001 for each). The majority of the examined subjects reached a cortisol peak, at a value greater than 18 μg/dl at 30 minutes. The mean cortisol levels at baseline were 8.2 μg/dl (95% CI 7.4-8.9) at 30 minutes 18.4 μg/dl (95% CI 17.2-19.7) and 21.2 μg/dl (95% CI 19.6-22.8) at 60 minutes. There was a strong positive correlation between 30 and 60 minutes cortisol values after Synacthen injection (Pearson r =0.83; p=0.0001). Further analysis using ROC curves revealed better diagnostic performance for cortisol after 30 minutes (AUC 30’ 0.865; 95% CI 0.788 – 0.941) in relation to 60 minutes after Synacthen administration (AUC 60’ 0.814; 95% CI 0.725 – 0.904) (**Figure 1**). Moreover, using 30 minutes cortisol level was resulting in only 9 false negative cases, in opposite to 30 or 60 minutes cortisol level where the number of FN was two times greater, 18 cases.

Additionally, we have compared different cut-off values for cortisol levels 30 minutes after Synacthen administration. Each cut-off value (18 μg/dl, 15.2 μg/dl, and 21.7 μg/dl; our cut off; advised by NHS Gloucestershire hospital and advised by Biochemical Investigations in Laboratory Medicine) was statistically significant, but only 18 μg/dl had the best overall performance **(** **Table 3** **).**

**Table 3:** Comparison of different cortisol cut off values in SST.

Analyzed group								
N=89	Cut off [µg/dl]	Se	Sp	NPV	PPV	ACC	RR (95% Cl)	p-value
30 minutes	18	76.9	82.0	82.0	76.9	79.8	4.3 (95% CI 1.8-10.04)	0.0000
30 minutes NHS Gloucestershire hospital	15.2	46.2	96.0	69.6	90	74.1	2.9 (95% Cl 1.3-6.6)	0.0000
30 minutes Biochemical Investigations in Laboratory Medicine	21.7	97.4	34.0	94.4	53.5	61.8	9.6 (95% Cl 1.2-74.9)	0.0001

Se, senistivity; Sp, specificity; NPV, negativepredictive value; PPV, positivepredictive value; ACC, accuracy;RR, risk ratio; 95%CI, 95%confidence interval; p-value for Fisher exact test.

### Accuracy of ACTH level in Metyrapone test in predicting SST results

**Figure 2:**
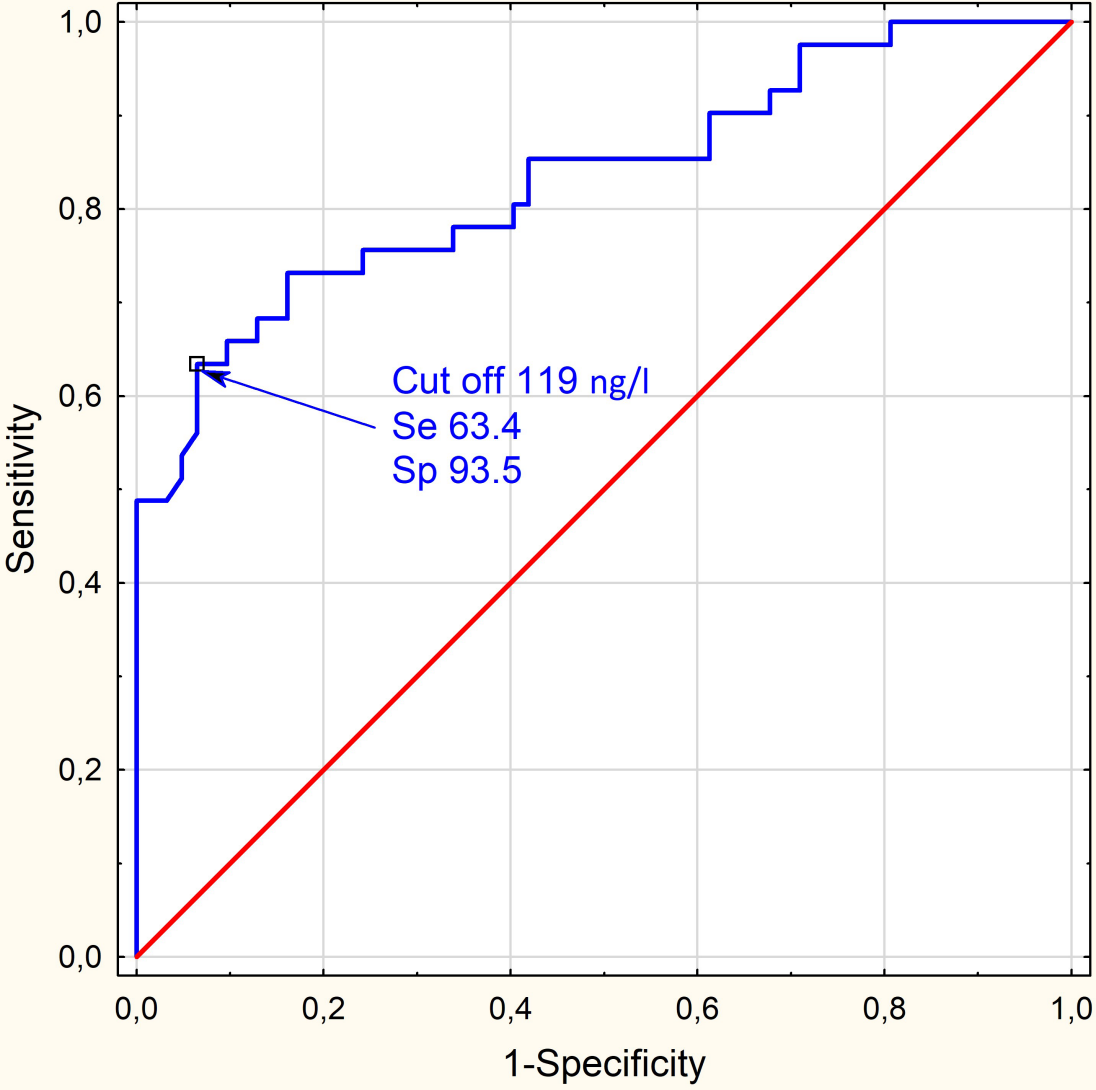
A ROC curve: ACTH<119 ng/l after Metyrapone had a sensitivity of 71.4% for predicting SAI (diagnosed in the SST) and specificity of 80.9% (AUC = 0.777; 95% CI 0.663 - 0.981; p<0.0001). N=103.

We performed ROC curve analyses with the aim of finding the ACTH cut-off level able to predict an accurate value for SAI (**Figure 2**).

Post-Metyrapone ACTH levels correlated moderately with the levels of cortisol after Synacthen administration in the analyzed 89 SSTs (Spearman r=0.44 p=0.0012). A ROC curve performed on this data set showed that ACTH<119 ng/l had a sensitivity of 71.4% for predicting failure of the SST (predicting SAI) and specificity of 80.9% for predicting passing the SST (AUC = 0.777; 95% CI 0.663 - 0.981; p=0.000). For this cut-off computed RR was 5.4 (95% CI 1.9 – 15.5), which means that patients with ACTH levels lower than 119 ng/l had 5.4 times the risk of SAI in SST compared to the group of patients who had ACTH levels at least 119 ng/l.

#### Comparison of performance of different ACTH cut-off values

**Figure 3:**
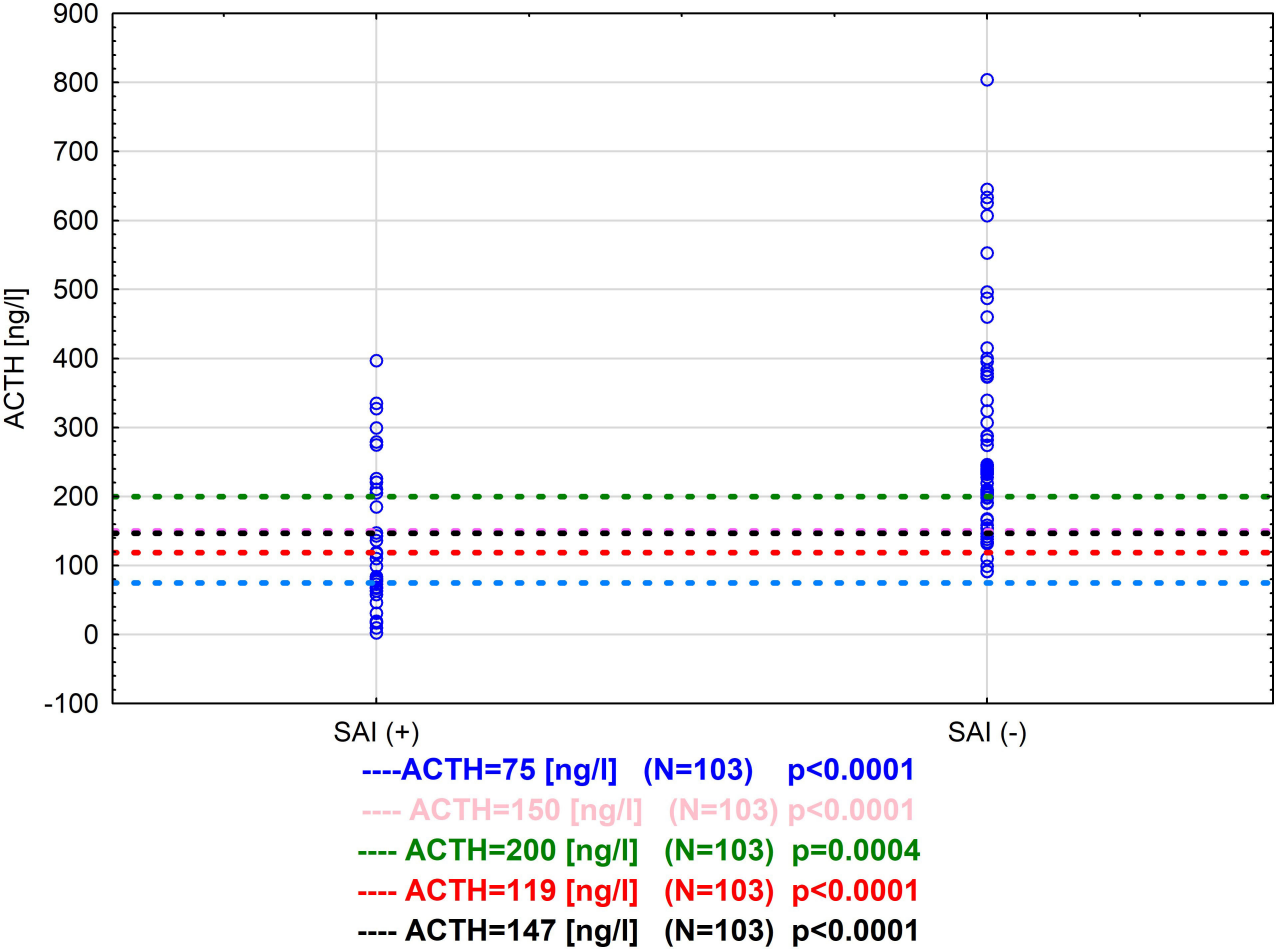
Comparison of different cut off values for ACTH in metyrapone test for our tested group (from literature and estimated in our patients) N=103.

In **Table 4** we are presenting a comparison of the performance of different cut-off values for ACTH in the metyrapone test. We decided to use three cut-offs found in the literature (75 ng/l, 150 ng/l, 200 ng/l) and our estimates based on ROC curves (119 ng/l and 147 ng/l) (shown on scatterplot **Figure 3**).

**Table 4:** Comparison of different ACTH cut off values (from literature and estimated in our patients).

Analyzed group							
N=103	Se	s	NPV	PPV	ACC	RR (95% Cl)	p-value
ACTH 75	36.6	100	70.5	100	74.8	3.4	0.0000
ACTH 150	73.2	83.9	82.5	75	79.6	4.3 (1.9-9.5)	0.0000
ACTH 200	73.2	69.4	79.6	61.2	70.9	3.0 (1.4.6)	0.0004
ACTH 119	63.4	93.5	79.5	86.7	81.6	4.2 (2-9.1)	0.0000
ACTH 147	73.2	83.9	82.5	75	79.6	4.3 (1.9-9.5)	0.0000

Se, senistivity; Sp, specificity; NPV, negativepredictive value; PPV, positivepredictive value; ACC, accuracy; RR, risk ratio; 95%CI, 95%confidence interval; p-value for Fisher exact test.

**Figure 4:**
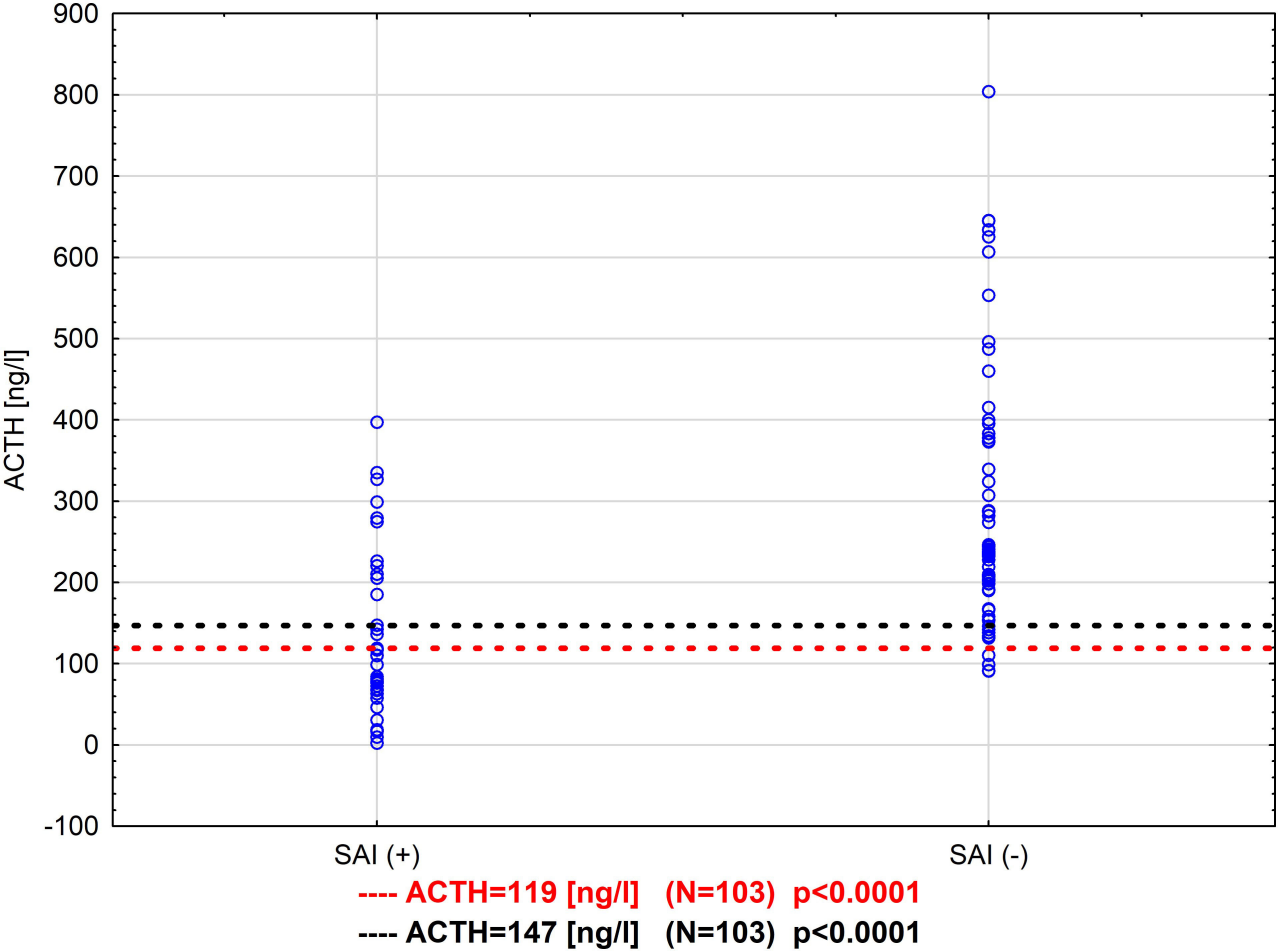
Comparison of different cut-off values for ACTH in the metyrapone test - two thresholds were found in two ways: SAI diagnosed by SST (ACTH = 119 ng/l) and by metyrapone test (ACTH = 147 ng/ml) N=103.

The highest sensitivity - 71.4% was noted for ACTH=147ng/l, 150 ng/l and 200 ng/l, while specificity was higher for both 147ng/l and 150ng/l in comparison with 200 ng/l 83.9% vs 69.4%. The highest specificity for predicting passing the metyrapone test had 75 ng/l 100%, but at the same time in 26 cases the test result would be false negative. The NPV, PPV, ACC and risk ratio for 147 ng/l, 150 ng/l and 119 ng/l were comparable 82.5%, 75.0%, 79.6% and 4.3 (95% CI 1.9-9.5) vs 79.5%, 86.7%, 81.6% and 4.2 (95% CI 1.9-9.1) respectively (**Table 4**). A comparison of estimated in this study cut-off values is presented in a scatterplot in **Figure 4**.

## Discussion

The proper diagnosis of adrenal insufficiency is essential In any case. Undiagnosed adrenal insufficiency or delayed diagnosis may lead to a life-threatening adrenal crisis (1, 2). On the other hand, unnecessary lifelong steroid therapy in patients with false positive diagnoses of adrenal insufficiency could be very harmful, as it can enhance cardiovascular risk increasing the possibility of premature death (8, 9, 24). All of our patients have been treated with hydrocortisone in the past or currently, some of them without significant improvement in their general condition and quality of life. After very thorough testing, we were able to confidently rule out adrenal insufficiency in as many as 63 of the 103 patients studied (61%).

In patients with secondary adrenal insufficiency, especially in states of incomplete ACTH deficiency, the “gold standard” - Synacthen short test can give false-negative results (25, 26). The use of 1 μg instead of 250 μg of Synacthen was initially thought to be a better procedure (more physiologic blood concentrations of 1-24ACTH (27), but some later studies have not confirmed this approach (28). In our material, there was a group of 18 patients who did not respond adequately to Metyrapon (their 11-deoxycortisol levels were below 7.0 μg/dl), although a diagnosis of adrenal insufficiency was initially ruled out after 1 and 250 μg of Synacthen. Such cases are reported in the literature and should be taken into account in the individual approach to the patient (23, 24).

This discrepancy between Synacthen and Metyrapon (or insulin) test results is mainly expected in patients in the first months after pituitary surgery (20). In our cases, this may be due to a partially preserved corticotropic reserve, when the adrenal cortex does not atrophy due to partial (but insufficient under stress) ACTH secretion. The adrenal glands then respond properly to high concentrations of 1-24ACTH.

Another proposed approach is to assess cortisol levels only at 30 minutes after Synacthen administration. In our study indeed we have found better diagnostic performance for cortisol after 30 minutes compared to 60 minutes after Synacthen administration. Using only 30 minute cortisol levels yielded only nine false-negative results, compared to 30 and 60 minute cortisol levels where the number of FN was two times greater. However, a retrospective study of as many as 804 patients with adrenal insufficiency did not find this difference (29). The appearance of false-negative SST results, regardless of how it was performed and interpreted, makes one remember the option of performing a test with Metyrapon - which for the diagnosis of secondary adrenal insufficiency may be more useful than Synacthen (30).

Testing with Metyrapone is fairly easy, but in our opinion requires hospitalization. Extremely low levels of cortisol in the blood can lead to malaise and even fainting before blood is drawn, so our patients are tested after one night in the hospital and in a prone position. In 50 healthy volunteers, tested on an outpatient basis, plasma ACTH rose to 64-907 ng/l by the Metyrapone test, however, 7 of them did not respond to the drug with an adequate reduction in serum cortisol levels (21). It was interpreted as probably due to incompliance with the intake of tablets (the drug was taken at home). In our study all 103 patients had adequate post-Metyrapone cortisol levels – good compliance is a bonus of hospitalization.

In none of the 103 subjects in the described group metyrapone caused serious side effects. However, the study is retrospective, and in our clinic if there are side effects of metyrapone, the test is stopped and the doctor on duty gives the patient 50 mg of hydrocortisone intravenously. Such a patient is therefore not included in the statistics, because he does not complete the test. After reviewing the records from 2016-2018, we found 2 patients in whom the test was discontinued due to nausea and vomiting after metyrapone administration.

In countries where metyrapone is not readily available, an alternative could be a test with osilodrostat, which acts at the same stage of cortisol synthesis as metyrapone. However, it has not yet been determined what dose of osilodrostat should be used with such a test. A very wide range of doses has been described for the treatment of hypercortisolemia with this new drug, so further research is needed to determine what dose should be used to achieve suppression of cortisol synthesis after a single administration of Osilodrostat.

The best ACTH cut-off value in SAI patients confirmed by the SST was surprisingly low (119 ng/l). This is most likely due to the fact that SST gives truly positive results in advanced disease, when the pituitary reserve is very significantly reduced. The best cut-off value of ACTH in relation to 11-deoxycortisol serum concentrations after Metyrapone administration was estimated at 147 ng/l - with a sensitivity of 73.2% and specificity of 83.9%. However, plasma ACTH was >200ng/l (the highest threshold proposed in literature) in 8 cases (20%) with a positive diagnosis of SAI made on the basis of low 11-deoxycortisole and confirmed with the short Synacthen test. Such results would be expected in patients with secondary adrenal insufficiency after treatment with high doses of glucocorticoids. In these cases, an increase in ACTH concentrations was observed first, and the adrenal function returns later (31). In our eight cases in which the 11-deoxycortisol response to Metyrapone was poor, despite high ACTH after Metyrapone, only three were after treatment with high doses of GCS, and two were after unilateral adrenalectomy due to hypercortisolemia (the same mechanism of secondary adrenal insufficiency as after GCS). In these patients, adrenal function did not return during follow-up. As the above summary and our experience show, fairly good results for sensitivity and specificity of the test fail in some cases, regardless of the cut-off adopted.

## Conclusions

The diagnosis of adrenal insufficiency should be carried out extremely carefully, and no single test seems to be conclusive for every case. Although the metyrapone test has proven to be a valuable tool in the diagnosis of secondary adrenal insufficiency, the determination of ACTH concentrations alone seems insufficient. A more difficult-to-obtain assessment of 11-deoxycortisole concentration values is necessary.

## Data availability statement

The original contributions presented in the study are included in the article/supplementary material. Further inquiries can be directed to the corresponding author.

## Ethics statement

The studies involving human participants were reviewed and approved by EC at Medical Center of Postgraduade Education. Written informed consent for participation was not required for this study in accordance with the national legislation and the institutional requirements.

## Author contributions

LP - conception of work, data collection, article writing; MR - consultations of work shedule and conception, patients management; BM - Statistical analyses; DL - conducting examinations in the clinic; KN - conducting examinations in the clinic; AŁ-S - conducting examinations in the clinic; PG - hormonal analyses; WZ - Head of Clinic. All authors contributed to the article and approved the submitted version.

## Conflict of interest

The authors declare that the research was conducted in the absence of any commercial or financial relationships that could be construed as a potential conflict of interest.

## Publisher’s note

All claims expressed in this article are solely those of the authors and do not necessarily represent those of their affiliated organizations, or those of the publisher, the editors and the reviewers. Any product that may be evaluated in this article, or claim that may be made by its manufacturer, is not guaranteed or endorsed by the publisher.

## References

[1] Bleicken B Hahner S Ventz M Quinkler M. Delayed diagnosis of adrenal insufficiency is common: a cross-sectional study in 216 patients. Am J Med Sci (2010) 339(6):525. doi: 10.1097/MAJ.0b013e3181db6b7a 20400889

[2] Papierska L Rabijewski M. Delay in diagnosis of adrenal insufficiency is a frequent cause of adrenal crisis. Int J Endocrinol (2013) 2013:482370. doi: 10.1155/2013/482370 23864857 PMC3707239

[3] Saevik ÅB Åkerman A-K Grønning K Nermoen I Valland SF Finnes TE. Clues for early detection of autoimmune addison's disease - myths and realities. J Intern Med (2018) 283(2):190–9. doi: 10.1111/joim.12699 29098731

[4] Hahner S Loeffler M Fassnacht M Weismann D Koschker AC Quinkler M. Impaired subjective health status in 256 patients with adrenal insufficiency on standard therapy based on cross-sectional analysis. J Clin Endocrinol Metab (2007) 92:3912–22. doi: 10.1210/jc.2007-0685 17684047

[5] Andrioli Pecori Giraldi F Cavagnini F. Isolated corticotrophin deficiency. Pituitary (2006) 9(4):289–95. doi: 10.1007/s11102-006-0408-5 17077949

[6] Nieman LK. Dynamic evaluation of adrenal hypofunction. J Endocrinol Invest (2003) 26(7 Suppl):74–82. 14604069

[7] Arlt W Allolio B. Adrenal insufficiency. THE Lancet (2003) 361:1881–93. doi: 10.1016/S0140-6736(03)13492-7 12788587

[8] Johannsson G Ragnarsson O. Cardiovascular and metabolic impact of glucocorticoid replacement therapy. Front Horm Res (2014) 43:33–44. doi: 10.1159/000360556 24943296

[9] CS H Danneberg S Harbeck B. Increased cardiovascular risk in patients with adrenal insufficiency: A short review. BioMed Res Int (2017) 2017:3691913. doi: 10.1155/2017/3691913 29376070 PMC5742446

[10] Speckart PF Nicoloff JT Bethune JE. Screening for adrenocortical insufficiency with cosyntropin (synthetic ACTH). Arch Intern Med (1971) 128:761–3. doi: 10.1001/archinte.1971.00310230091007 4330323

[11] Raff H Sharma ST Nieman LK. Physiological basis for the etiology, diagnosis, and treatment of adrenal disorders: Cushing’s syndrome, adrenal insufficiency, and congenital adrenal hyperplasia. Compr Physiol (2014) 4(2):739–69. doi: 10.1002/cphy.c130035 PMC4215264 24715566

[12] Neary N Nieman LK. Adrenal insufficiency- etiology, diagnosis and treatment. Curr Opin Endocrinol Diabetes Obes (2010) 17(3):217–23. doi: 10.1097/MED.0b013e328338f608 PMC2928659 20375886

[13] Crowley RK Argese N Tomlinson JW Stewart PM. Central hypoadrenalism. J Clin Endocrinol Metab (2014) 99:4027–36. doi: 10.1210/jc.2014-2476 25140404

[14] Petersenn S Quabbe HJ Schofl C Stalla GK von Werder K Buchfelder M. The rational use of pituitary stimulation tests. Dtsch Arztebl Int (2010) 107:437–43. doi: 10.3238/arztebl.2010.0437 PMC2905884 20644702

[15] Maghnie M Uga E Temporini F Di Iorgi N Secco A Tinelli C. Evaluation of adrenal function in patients with growth hormone deficiency and hypothalamic–pituitary disorders: comparison between insulin-induced hypoglycemia, low-dose ACTH, standard ACTH and CRH stimulation tests. EJE (2005) 152:735–41. doi: 10.1530/eje.1.01911 15879359

[16] Lopez Schmidt I Lahner H Mann K Petersenn S. Diagnosis of adrenal insufficiency: Evaluation of the corticotropin-releasing hormone test and basal serum cortisol in comparison to the insulin tolerance test in patients with hypothalamic-Pituitary-Adrenal disease. J Clin Endocrinol Metab (2003) 88:4193–8. doi: 10.1210/jc.2002-021897 12970286

[17] Steiner H Bähr V Exner P Oelker PW. Pituitary function tests: comparison of ACTH and 11-deoxycortisol responses in the metyrapone test and with the insulin hypoglycemia test. Exp Clin Endocrinol (1994) 102:33–8. doi: 10.1055/s-0029-1211262 8005206

[18] Fiad TM Kirby JM Cunningham SK MCKenna TJ. The overnight single dose metyrapone test is a simple and reliable index of the hypothalamic-pituitary-adrenal axis. Clin Endocrinol (1994) 40:603–9. doi: 10.1111/j.1365-2265.1994.tb03011.x 8013141

[19] Endert E Ouwehand A Fliers E Prummel MF Wiersinga WM. Establishment of reference values for endocrine tests. part IV : Adrenal insufficiency. Netherlands Jpurnal Med (2005) 63(11):435–43. 16397312

[20] Karaca Z Grossman A Kelestimur F. Investigation of the hypothalamo-pituitary-adrenal (HPA) axis: a contemporary synthesis. Rev Endocr Metab Disord (2021) 22(2):179–204. doi: 10.1007/s11154-020-09611-3 33770352

[21] Yeo KT Babic N Hannoush ZC Weiss RE. Endocrine testing protocols: Hypothalamic pituitary adrenal axis . Available at: https://www.ncbi.nlm.nih.gov/books/NBK278940. 25905177

[22] Rosenberg AGW Pellikaan K Poitou C Goldstone AP Høybye C Markovic T. Central adrenal insufficiency is rare in adults with prader-willi syndrome. J Clin Endocrinol Metab (2020) 105(7):1–9. doi: 10.1210/clinem/dgaa168 32232324 PMC7211032

[23] Metopirone - summary of product characteristic . Available at: https://www.hpra.ie/img/uploaded/swedocuments/Licence_PA22888-001-001_02062020081848.pdf.

[24] Bergthorsdottir R Leonsson-Zachrisson M Odeín A Johannsson G. Premature mortality in patients with addison’s disease: A population-based study. J Clin Endocrinol Metab (2006) 91(12):4849–53. doi: 10.1210/jc.2006-0076 16968806

[25] Streeten D Anderson G Bonaventura M. The potential for serious consequences from misinterpreting normal responses to the rapid adrenocorticotropin test. J Clin Endocrinol Metab (1996) 81:285–90. doi: 10.1210/jcem.81.1.8550765 8550765

[26] Blum CA Schneeberger D Lang M Rakic J Michot MP Müller B. Acute-onset panhypopituitarism nearly missed by initial cosyntropin testing. Case Rep Crit Care (2017) 2017:7931438. doi: 10.1155/2017/7931438 29109870 PMC5646303

[27] Tordjman K Jaffe A Grazas N Apter C Stern N. The role of the low dose (1mg) adrenocorticotropin test in the evaluation of patients with pituitary diseases. J Clin Endocrinol Metab (1995) 80:1301–5. doi: 10.1210/jcem.80.4.7714104 7714104

[28] Ceccato F Scaroni C. Central adrenal insufficiency: open issues regarding diagnosis and glucocorticoid treatment. Clin Chem Lab Med (2019) 57(8):1125–35. doi: 10.1515/cclm-2018-0824 30427776

[29] Struja T Briner L Meier A Kutz A Mundwiler E Huber A. Diagnostic accuracy of basal cortisol level to predict adrenal insufficiency in cosyntropin testing: Results from an observational cohort study with 804 patients. Endocr Pract (2017) 23(8):949–61. doi: 10.4158/EP171861.OR 28614010

[30] Soule S Van Zyl Smit C Parolis G Attenborough S Peter D Kinvig S. The low dose ACTH stimulation test is less sensitive than the overnight metyrapone test for the diagnosis of secondary hypoadrenalism. Clin Endocrinol (Oxf) (2000) 53(2):221–7. doi: 10.1046/j.1365-2265.2000.01057.x 10931104

[31] Cunha CF Silva IN Finch FL. Early adrenocortical recovery after glucocorticoid therapy in children with leukemia. J Clin Endocrinol Metab (2004) 89(6):2797–802. doi: 10.1210/jc.2003-031165 15181060

